# Long-term disease control in dedifferentiated liposarcoma: a case report on trabectedin priming followed by PD-1 inhibition

**DOI:** 10.3389/fonc.2024.1518775

**Published:** 2025-01-16

**Authors:** Johannes M. Waldschmidt, Lukas Haug, Christine Riedhammer, Christoph K. W. Deinzer, Marcus Zimmermann, Anke Heidemeier, Peter Raab, Maximilian Rudert, Anne Hendricks, Johan F. Lock, Viktoria Buck, Andreas Rosenwald, Hermann Einsele, Peter Reichardt, Volker Kunzmann, Armin Wiegering, Daniel Pink, K. Martin Kortüm

**Affiliations:** ^1^ Department of Internal Medicine II, University Hospital Würzburg, Würzburg, Germany; ^2^ Bavarian Cancer Research Centre (BZKF), Würzburg, Germany; ^3^ Department of Pathology, University Hospital Würzburg, Würzburg, Germany; ^4^ Department of Internal Medicine VIII, University Hospital Tübingen, Tübingen, Germany; ^5^ Department of Radiation Therapy, University Hospital Würzburg, Würzburg, Germany; ^6^ Department of Radiology, University Hospital Würzburg, Würzburg, Germany; ^7^ Department of Orthopaedic Surgery, König Ludwig Haus, University of Würzburg, Würzburg, Germany; ^8^ Department of Visceral Surgery, University Hospital Würzburg, Würzburg, Germany; ^9^ Department of Oncology, Helios Klinikum Berlin-Buch, Berlin, Germany; ^10^ Department of Oncology and Palliative Care, Helios Klinikum Bad Saarow, Bad Saarow, Germany; ^11^ Klinik und Poliklinik für Innere Medizin C, Universitätsmedizin Greifswald, Greifswald, Germany

**Keywords:** sarcoma, dedifferentiated liposarcoma, checkpoint inhibition, PD-1, immune microenvironment

## Abstract

**Background:**

Dedifferentiated liposarcoma (DDLPS) is a rare mesenchymal cancer originating from the adipose tissue, with poor survival rates for most patients, highlighting the critical need for novel treatment options.

**Case description:**

This report examines the efficacy and safety of sequential pre-treatment with the marine-derived alkaloid trabectedin followed by checkpoint inhibition using the anti-PD-1 antibody nivolumab in a 63-year-old male patient with unresectable retroperitoneal DDLPS. Treatment was initiated at the time of the seventh relapse as part of the NitraSarc phase 2 multicenter trial for inoperable soft tissue sarcoma conducted by the German Interdisciplinary Sarcoma Group (GISG-15, *NCT03590210*). The patient demonstrated an immediate tumor response, and in combination with minor surgery, achieved R0 resection status, which was subsequently maintained without the need for further therapy for the past 52 months. Correlative molecular analyses revealed a sustained DNA damage repair machinery and downregulation of PD-1 protein expression in post-treatment tumor samples.

**Conclusion:**

This report provides exemplary insight on the feasibility and efficacy of sequential pre-treatment with trabectedin as a priming strategy for PD-1 inhibition in advanced DDLPS. Full trial results from NitraSarc are pending for publication.

## Introduction

Dedifferentiated liposarcoma (DDLPS) is a rare but aggressive variant of soft-tissue sarcoma (STS) that can occasionally arise in the retroperitoneum, accounting for approximately 10% of all liposarcomas (LPS) (1). Therapeutic strategies for DDLPS generally employ a multimodal approach, including primary surgery, radiation therapy, and anthracycline-based chemotherapy (2). Although novel targeted therapies have been introduced, they have yet failed to demonstrate an improvement in overall survival (OS) and prognosis for DDLPS patients remains poor, with a 5-year OS estimated at around 30%. This underscores the critical need for innovative therapeutic strategies to improve clinical outcomes (3).

In recent years, immune checkpoint inhibitors targeting the programmed death receptor 1 (PD-1) and cytotoxic T-lymphocyte-associated protein 4 (CTLA-4) have significantly advanced the treatment of many solid tumors, sparking growing interest in exploring their role in DDLPS. Several clinical trials have investigated the efficacy of anti-PD-1 monoclonal antibodies in selected STS subtypes, administered as monotherapy (4, 5) or in combination with chemotherapeutics (6, 7), targeted agents (8, 9), or anti-CTLA-4 antibodies (10, 11). These studies however have shown limited activity (**Table 1**), indicating that further research is warranted to better understand the molecular characteristics and optimal combination strategies that may enhance treatment efficacy.

**Table 1:** Review of clinical trials investigating immune checkpoint inhibition in advanced soft-tissue sarcoma.

Treatment	n	Design	Dosing	Response	Ref
Nivolumab (N) + Trabectedin (Trab)	STS: n=92 LPS: n=15	phase-2	Trab: 1.5mg/m² q3wk + N: 240mg q2wk (≥c4:LCC, ≥c2:ECC)	6-mos-PFS: 48% (LPS, LMS) mPFS 5.5 mos (LPS, LMS)	(12)
Nivolumab (N)	STS: n=21 LPS: n=8	phase-2	N: 240mg q2wk	ORR: 0% mPFS: 1.4 (1.4-2.8) mos 6-mos-OS: 86%	(5)
Pembrolizumab (P)	STS: n=80 LPS: n=10	phase-2	P: 200mg q3wk	ORR: 20% (LPS) CBR: 60% (LPS) mPFS: 6.3 mos (LPS)	(4)
Pembrolizumab (P) + doxorubicin (Dox)	STS: n=37 LPS: n=5	phase-1/2	P: 200mg q3wk + Dox: 45 vs. 75mg/m^2^ q3wk (c2-7)	ORR: 19% CBR: 54% mPFS: 8.1 mos 12-week PFS: 81%	(6)
Pembrolizumab (P) + cyclophosphamide (Cy)	STS: n=57 LPS: n=5	phase-2	P: 200mg q3wk + Cy: 50mg BID (1wk on/off)	ORR: 6% mPFS: 1.4 mos	(7)
Pembrolizumab (P) + epacadostat (E)	STS: n=30 LPS: n=3	phase-2	P: 200mg q3wk + E: 100mg BID	ORR: 3% mPFS: 1.7 mos	(8)
Pembrolizumab (P) + eribulin (Eri)	STS: n=57 LPS: n=20	phase-2	P: 200mg q3wk + Eri: 1.4mg/m^2^ (d1 + 8)	ORR: 15% (LPS) CBR: 75% (LPS) 12-week PFS: 70% (LPS)	(9)
Durvalumab (D) + tremelimumab (T)	STS: n=57 LPS= n=6	phase-2	c1-4: D: 1500mg + T: 75mg q4wk ≥c5: D: 1500mg q4wk	ORR 12% 12-week PFS: 49%	(10)
Durvalumab (D) + tremelimumab (T) vs. doxorubcin (Dox)	STS: n=53 vs. n=39	phase-3	D: 1500mg q4wk + T: 75mg 3x q4wk (≥c4 q12wk) vs. Dox 75mg/m^2^ 6x q3wk	ORR: 9% vs. 13% mPFS 2.7 vs. 2.8 mos mOS: 17.4 vs. 12,5 mos	(11)

STS, soft-tissue sarcoma; LPS, liposarcoma; LMS, leiomyosarcoma; q2wk, every two weeks; BID,twice daily; LCC, late combination cohort; ECC, early combination cohort; mPFS, median progression-free survival; mos, months; ORR, overall response rate; CBR, clinical benefit rate; mOS, median overall survival.

In this case study, we investigated the hypothesis that pre-treatment with the marine-derived alkaloid trabectedin could prime the sarcoma microenvironment for enhanced responsiveness to subsequent PD-1 inhibition in a patient with advanced and unresectable DDLPS.

## Case description

**Figure 1:**
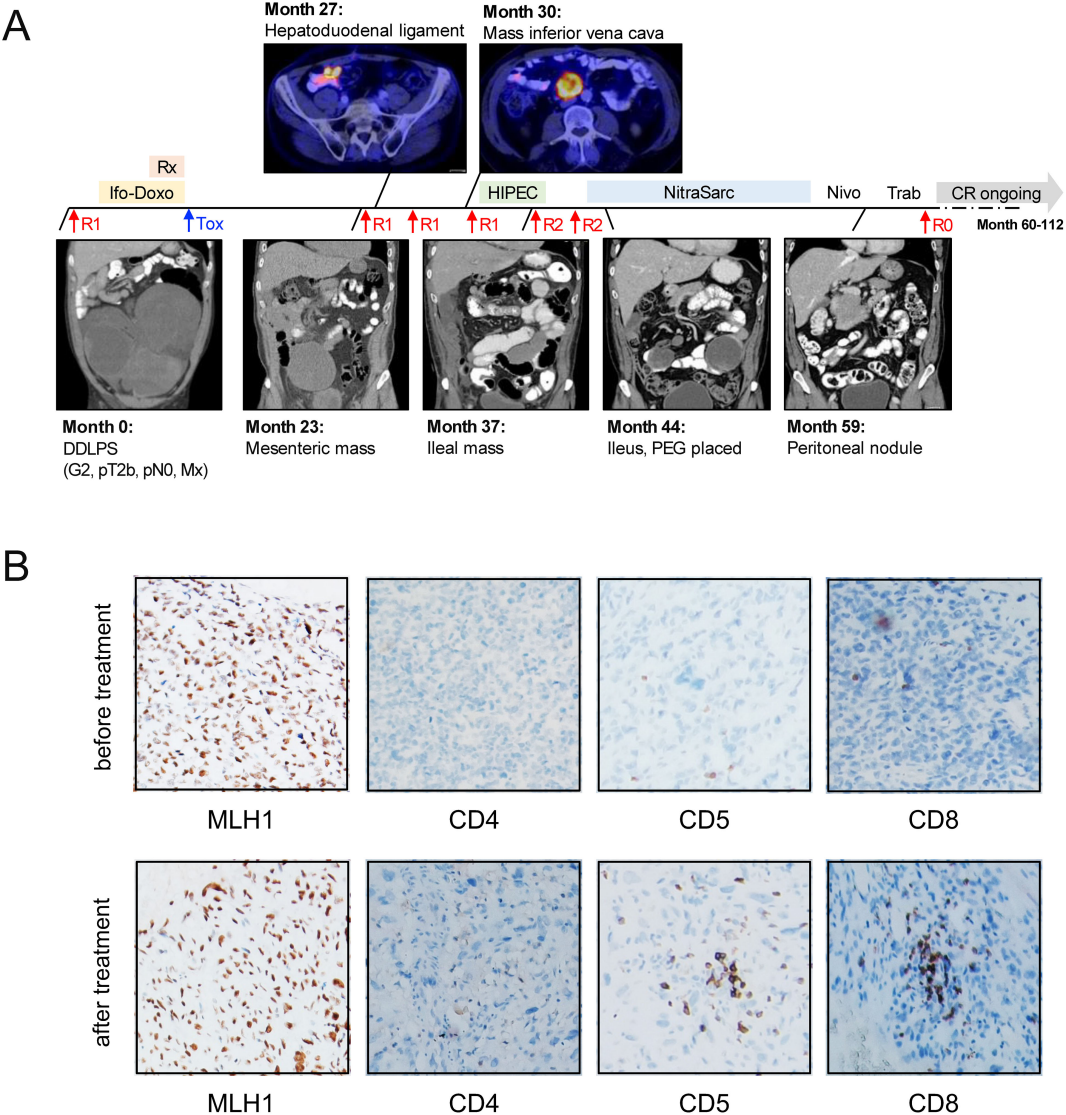
Clinical course and molecular assessment in a 63-year old patient with DDLPS. **(A)** Graphical summary of the patient history. Over the course of 112 months, the patient underwent a total of seven surgical procedures. Trabectedin therapy, followed by nivolumab treatment, was initiated as a palliative measure at month 43. This approach, combined with a minor tumor resection via jejuno-jejunostomy, resulted in an R0 resection, representing the first successful R0 status achieved 60 months after the initial diagnosis. The patient has since remained without evidence of disease. **(B)** IHC staining for MLH1 (left, 100x) and CD4, CD5 and CD8 (right, 40x) in representative specimens before and after treatment with trabectedin and nivolumab. Rx, radiation therapy; Ifo-Doxo, ifosfamide-doxorubicin; Tox, hematotoxicity; R0/R1/R2, surgery performed resulting in R0/R1/R2 resection status; HIPEC, intraperitoneal hyperthermic chemoperfusion; Nivo, nivolumab; Trab, trabectedin; CR, complete remission; PEG, percutaneous endoscopic gastrostomy.

We investigated this rationale in a 63-year old male patient diagnosed with DDLPS of the retroperitoneum, who subsequently underwent upfront surgery resulting in an R1 resection (**Figure 1A**). The tumor was classified as pT2b, pN0, M0 according to TNM classification, and FNCLCC grade 3 + 3 + 0 = 6 with confirmed positivity for MDM2 and CDK4 by immunohistochemistry (IHC). The patient received adjuvant chemotherapy with ifosfamide and doxorubicin, along with radiation therapy; however, this treatment had to be discontinued after three cycles due to severe dose-limiting hematotoxicity. The patient remained in remission until the first relapse at 23 months. During this relapse, resection of a tumor mass proximal to the mesenteric root led to R1 status and prompted a second-look laparotomy, which involved wedge resection of the cecal wall, partial resection of the peritoneum, and removal of a tumorous lymph node from the hepatoduodenal ligament.

At month 26, a novel mass was detected on the ventral side of the inferior vena cava. The patient underwent tumor debulking and hyperthermic intraperitoneal chemotherapy (HIPEC), however, a third abdominal relapse occurred shortly after at month 32. This led to a fifth and sixth debulking surgery, which, despite partial resection of the ileum and ileo-transversostomy, resulted in an R2 resection status. At this point, palliative options were thoroughly discussed with the patient, and it was decided to initiate study treatment within the NitraSarc trial, a phase-2 multicenter study for inoperable soft-tissue sarcoma conducted by the German Interdisciplinary Sarcoma Group (GISG-15, *NCT03590210) (* 12).

In this trial, trabectedin was administered at a dose of 1.5 mg/m² over 24 hours every three weeks (q3w) for cycles 1-3, with nivolumab (240 mg) added on the same schedule starting from cycle 4. During cycle 1, while still receiving single-agent trabectedin, the patient developed clinical signs of a mechanical ileus, necessitating the placement of a venting percutaneous endoscopic gastrostomy for palliation. Despite this setback, and recognizing the lack of alternative treatment options, the patient continued with the study therapy, leading to rapid improvement in clinical symptoms. CT staging revealed disease stabilization with a slight tumor regression of -26% after three cycles of single-agent trabectedin. Following an additional four cycles of combined trabectedin and checkpoint inhibition, the response deepened to a partial response (-86%) according to RECIST 1.1.

Throughout subsequent treatment cycles, the patient continued to benefit from the therapy. Trabectedin was discontinued after eleven cycles due to ongoing infectious complications. Given the excellent remission status at that point, we decided to continue with single-agent nivolumab q3w as a maintenance strategy. After three more cycles, CT staging detected a new tumorous nodule within the peritoneum. In response, single-agent trabectedin was re-started, and a re-laparotomy with tumor resection via jejuno-jejunostomy was performed. These interventions resulted in an R0 resection, marking the first successful R0 status achieved at 60 months post-initial diagnosis. In light of this outstanding result, no additional treatment was initiated. The patient has currently no evidence of disease (NED) and has been living with DDLPS for 9.3 years, including the past 4.3 years without any treatment.

## Discussion

Our experience on the efficacy and durability of combined trabectedin and checkpoint inhibition in a patient with advanced and unresectable DDLPS complements prior findings from the SARC028 study (*NCT02301039*). This phase-2 clinical trial investigated the efficacy of the anti-PD-1 monoclonal antibody pembrolizumab in patients with metastatic or surgically inoperable, locally advanced soft tissue sarcoma (n=80, including n=10 with LPS) (4). In the SARC028 trial, only partial responses were observed in a minority of LPS patients (2/10 = 20%), with a median progression-free survival (PFS) of 25 weeks and a 12-week PFS of 60% across the entire subgroup. Initial results from another phase-2 study, evaluating the anti-PD-1 monoclonal antibody nivolumab in 23 patients (including n=8 with LPS), reported no clinical responses, resulting in a poor median PFS of only 1.4 months (5). Preliminary efficacy data from the NitraSarc trial were recently disclosed in an interim analysis after a median follow-up of 16.6 months (12). The median PFS in this analysis was 5.5 months in the LPS and leiomyosarcoma subgroup, compared to 2.3 months in patients with non-L-sarcomas (pleomorphic, spindle cell, fibromyxoid, synovial, and epithelial sarcomas), with corresponding median OS of 18.7 and 5.6 months, respectively. While these preliminary results do not support further investigation of trabectedin/nivolumab in non-L-sarcomas, it is noteworthy that the presented case demonstrated an exceptional response duration, even within the L-sarcoma subgroup.

Given that most LPS patients do not respond to checkpoint inhibition, these findings underscore the unmet clinical need to better characterize patients with anti-PD-1-responsive subtypes. In the SARC028 trial, objective responses were exclusively observed in patients with high PD-1 expression on tumor cells and tumor-associated macrophages (4). It has been suggested that soft-tissue sarcomas with an unbalanced karyotype and high mutational load may elicit more effective immune responses (13). Additionally, genetically complex sarcomas like DDLPS are often characterized by the expression of multiple neoantigens, which can enhance responses from tumor-infiltrating T cells (14). Both the re-exposure to neoantigens and the eradication of M2 macrophages within the tumor microenvironment have been proposed as potential mechanisms associated with trabectedin therapy in sarcoma patients (15), possibly contributing to the therapeutic effect observed in our patient, and warrant validation as prospective biomarkers for trabectedin/checkpoint inhibitor response.

In alignment with these findings, a recent study conducted by the Cancer Genome Atlas (TCGA) consortium indicated that overexpression of PD-1 and PD-L1, along with an increased number of infiltrating T and NK cells, may predict a favorable response to checkpoint inhibition (16). Furthermore, an immune infiltration score, characterized by a high level of copy-number aberrations, increased interferon-gamma signaling, and the expression of exhaustion markers such as TIM-3, has recently been established to facilitate standardized screening for patients with checkpoint inhibitor-responsive disease.

As in our patient neither data on mutational burden nor next-generation sequencing were available, we performed IHC analysis of representative specimen before and after trabectedin treatment which both showed retained expression for the mismatch repair proteins MLH1, PMS1, MSH2 and MSH6. Furthermore, we characterized the rather mild inflammatory infiltrate, which showed increased T-lymphocytes (CD5/CD3 positive) near the invasion front of the tumor, whereas centrally in the tumor only few T-lymphocytes could be found. These T-lymphocytes were mostly characterized by high CD8 but low CD4 expression. Representative images for MLH1, CD4, CD5 and CD8 stains are displayed in **Figure 1B**. PD-1 staining revealed a minor fraction of intratumoral PD1 positive lymphocytes. 2% of tumor cells were positive for PD-L1 before versus <1% after treatment, this potentially conferring to the treatment efficacy observed in our patient. Due to spatial heterogeneity within the biopsies, quantification of T-lymphocytes was generally difficult and corresponded to a maximum effector:target ratio of 1:20 cells at both time points.

While our report presents valuable findings, it is important to acknowledge factors that may have contributed to the excellent outcome observed in our patient. Notably, our patient did not have distant metastases and was able to receive advanced treatment options, including HIPEC therapy, which are not available at many institutions. Moreover, our patient had only undergone three cycles of doxorubicin and ifosfamide without the addition of other chemotherapeutic agents. This limited exposure may have conferred a higher sensitivity to chemotherapy at the time of trabectedin treatment as compared to many other cases of DDLPS. However, this observation also highlights the potential benefit of administering trabectedin earlier in the treatment course, possibly as part of a maintenance strategy following doxorubicin-trabectedin based induction therapy. This approach is supported by recent findings from the pivotal phase-3 LMS-04 trial (*NCT02997358*), which demonstrated a median OS benefit of 33 vs. 24 months (hazard ratio 0.65, 95% CI, 0.44-0.95) for trabectedin-doxorubicin as compared to single-agent doxorubicin in chemotherapy-naïve patients with metastatic leiomyosarcoma (17). It is worth noting that the recent patent expiration of trabectedin may also increase accessibility for such strategies to provide improved efficacy while maintaining cost effectiveness.

## Conclusion

In summary, there remains an unmet clinical need for enhanced molecular characterization to more accurately identify sarcoma patients likely to benefit from checkpoint inhibitor-based immunotherapies. While the final results of the NitraSarc trial are still pending publication, our findings demonstrate the feasibility and potential efficacy of sequential pre-treatment with trabectedin followed by PD-1 inhibition in achieving long-term disease control in a patient with advanced and unresectable DDLPS.

## Data Availability

The original contributions presented in the study are included in the article/supplementary material. Further inquiries can be directed to the corresponding author.
